# Expression of matrix Metalloproteinases-2 and aquaporin-1 in corneoscleral junction after angle-closure in rabbits

**DOI:** 10.1186/s12886-019-1058-5

**Published:** 2019-02-04

**Authors:** Yaqin Jiang, Canwei Zhang, Jianli Ma, Luping Wang, Jing Gao, Jiantao Ren, Wei He, Sheng Wang, Shuai Sheng, Xudong Huang

**Affiliations:** 1 Department of Ophthalmology, Weifang Eye Hospital, Weifang, Shandong People’s Republic of China; 2 Department of Ophthalmology, University of Bonn, Bonn, Germany

**Keywords:** Angle-closure, Matrix metalloproteinases-2, Aquaporin-1, Trabecular meshwork, Schlemm’s canal

## Abstract

**Background:**

To investigate the expression of Matrix Metalloproteinases 2 and aquaporin-1 in corneoscleral junction and explore the mechanism of trabecular damageafter angle-closure.

**Methods:**

Thirty New Zealand white rabbits were randomly assigned into 2 groups, theexperimental group (Group 1) including twenty five rabbits and the control group (Group 2) including 5 rabbits. The rabbits in the experimental group were used to establish angle-closure models, and the rabbits in the control group were not subjected to any operation. All the rabbits were followed by slit lamp microscopy, Tonopen tonometer, and anterior segment optical coherent tomography (AS-OCT). The expressions of metalloproteinase MMP-2, aquaporin-1, and tissue inhibitors of metalloproteinase-2 in corneoscleral junctionwere evaluatedin both groups byimmunofluorescence, quantitative reverse-transcription polymerase chain reaction (qRT-PCR), and enzyme-linked immunosorbent assay (ELISA).

**Results:**

Slit-lamp examination showed that angle-closure model was successfully established in twenty rabbits. The extent of angle-closure was about 2 to 4 clock hours in all the rabbit models, but the intraocular pressure (IOP) of the rabbits distributed from 8.57 to 15.25 mmHg and no significant high IOP was found in the follow-up period. The AQP-1-positive cells mainly located in Schlemm’s canal, the inner surface of trabecular meshwork (TM), and the surface of iris, which began to decline on 1 month after angle-closure. MMP2 staining was diffuse in trabecular meshwork and iris. Immunofluorescence signal of MMP2 was strong within 1 month after angle-closure, and subsequently became weak. qRT-PCR and ELISA showed that the expression of MMP-2 and TIMP-2 increased within 1 month after angle-closure and then declined gradually. The AQP-1 levels showed slightly declined on 1 month after angle-closure.

**Conclusions:**

Altered levels of MMPs, TIMPs, and AQP-1 were found in the area of angle-closure, which may be involved in the damage of TM and Schlemm’s canal after angle-closure.

## Background

Angle-closure glaucoma (ACG) is a major cause of blindness worldwide and is characterized by increased intraocular pressure (IOP) due to appositional or synechial angle closure associated with visual field defects [1]. Goniosynechialysis has been reported to be an effective therapy for chronic angle-closure glaucoma by separation of peripheral anterior synechiae and showed fewer postoperative complications [2]. However, this surgery is limited to patients whose duration of synechial closure is not too long [3]. Long-standing peripheral anterior synechiae (PAS) may reduce the IOP-lowering effect of the surgery because of irreversible trabecular damage [4]. Previous studies showed that the generationof aqueous outflow resistance is most significant in the outer layer of the trabecular meshwork (TM) and innerwall endothelium of Schlemm’s canal (SC) [5]. The extracellular matrix(ECM) composition in the juxtacanalicular connectivetissue (JCT) region has been shown to particularly influenceoutflow patterns and resistance generation [6, 7]. Abnormal accumulations of ECM within the JCT have been identified as one of the main causes of the increasing IOP in primary open-angle glaucoma (POAG). Studies regarding the changes of TM and SC after angle-closure may hold a great significance for revealing the pathogenesis of ACG.

MMPs constitute part of a superfamily of metalloproteinases with conserved catalytic domain which become activated upon cleavage. MMPs have a vital role in ECM degradation and remodeling in the TM, which maintains the outflow pathway and IOPhomeostasis [8]. MMP-2 and -9 have reported to be associated with theoccurrence and development of glaucoma [9]. MMP-2 and MMP-9 degrade similar substrates, such as gelatin, collagen types IV and V, elastin, laminin, fibronectin, and proteoglycans. MMP-2 is primarily produced by stromal cells, including fibroblasts, myofibroblasts, and endothelial cells, and MMP-9 is mainly produced by neutrophils and to a lesser extent by eosinophils, monocytes, macrophages, lymphocytes, and epithelial cells [10]. It was reported that MMP-2 levels were increased in POAG cases [7]. In addition, MMP-2 plays an important role in TGFβ-mediated posterior capsule opacification formation [11]. However, the change of MMP-2 level after angle-closure was still not reported.

The regulation of aqueous volume and pressure is complicated and is a function of fluid exiting and entering the eye. Defects in this regulation may lead to the increase of IOP associated with glaucoma [12]. Previous study showed that aquaporin molecules are basically involved in water movement in tissues [13]. The aqueous humour must cross a bilayer of endothelial cells from entering the Schlemm’s canal to leaving the eye. Aquaporin-1(AQP-1) was reported to be expressed in Schlemm’s canal, TM, and iris, which plays an important role in the movement of water out of the eye [12]. However, after angle-closure happening, the expression of AQP-1 in Schlemm’s canal and TM was still unclear.

Based on all of these researches, in this study we detected the expression of MMP-2 and AQP-1 in corneoscleral junctionand explored the functional changes of Schlemm’s canal and TM after angle-closure. Moreover, previous study showed that increased co-localization of ECM proteins with endoplasmic reticulum stress markers was observed in human post-mortem glaucomatous TM tissues [14]. Therefore, the expression of MMP-2 and AQP-1 in rabbit’s eyes may also change after enucleation.In order to avoiding the potiental influence of enucleation on the expression of MMP-2 and AQP-1, the samples were immediately fixed in 4% formaldehyde or proceeded to protein and RNA extraction,

## Materials and methods

### Animals

**Fig.1:**
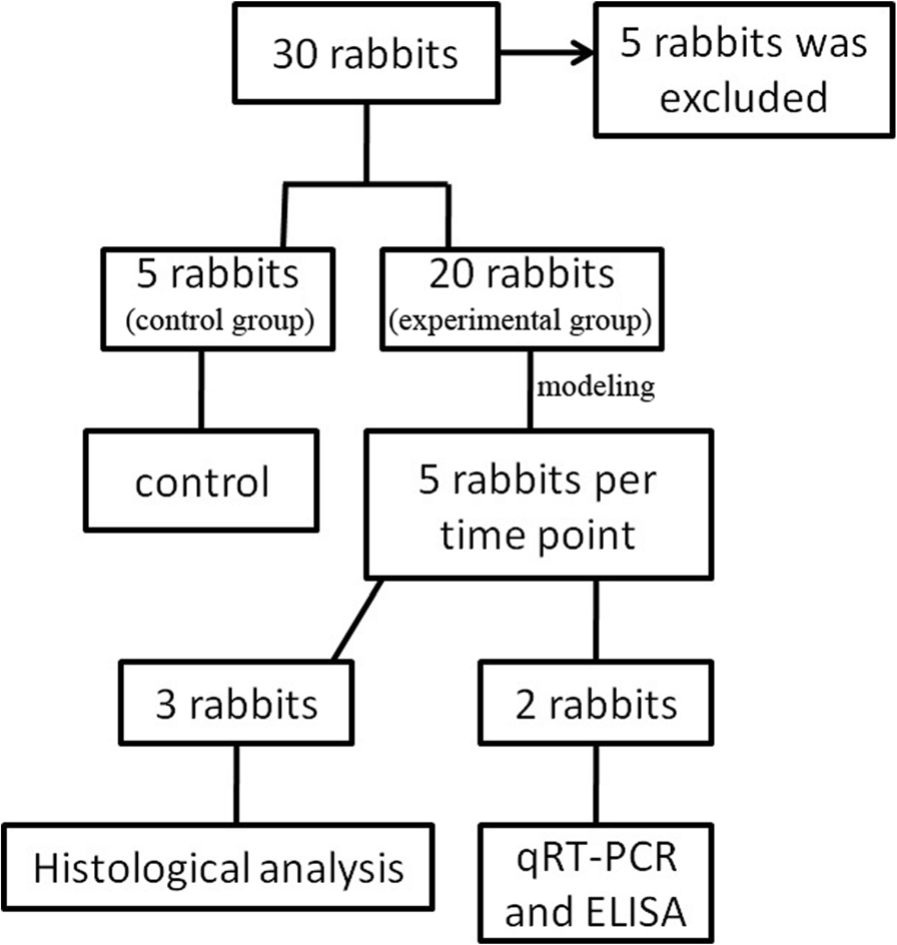
A flow chart about what happens to the experimental and control animals

Thirty male New Zealand white rabbits (Xilingjiao experimental animal breeding center, Jinan, China) weighing 2.0–2.5 kg (4 to 5-month-old) were used for this study (Fig. 1). All animals were treated in accordance to the ARVO (Association for Research in Vision and Ophthalmology) Statement for the Use of Animals in Ophthalmic and Vision Research, and the animal experiments were approved by the Medical Ethics Committee of Weifang Eye Hospital (The Medical Ethics Committee of Weifang Eye Hospital is a clinical research and animal ethics committee of China).

### Establishment of angle-closure model

Twenty five rabbits were used to produce angle-closure modelsas the following procedure. The rabbits were anesthetizedintravenously with 3% pentobarbital sodium (40–50 mg/kg) andtopically with oxybuprocaine hydrochloride. Three days before surgery, chloramphenicol eye drops were used 4 times per day. The pupil was dilatedwith tropicamide and phenylephrine eye drops. The incision was fabricated in corneal limbus, and aqueous humour was aspirated with 1 ml syringes until flat anterior chamber (corneo-lenticular touch). Sodium hyaluronate with the same volume of aspirated aqueous humour was injected into vitreous cavity at 3.5 mm back from the limbus. Ofloxacin ophthalmic gel was immediately used after surgery, and chloramphenicol eye drops were applied eight times per day. If angle-closure was not formed, the above steps were performed one more time. Another five normal rabbits without any operation were used as control. The rabbits were followed on postoperative 3 days, 1 week, 1 month, 2 and 4 months using slit lamp microscopy, Tonopen tonometer, and anterior segment optical coherent tomography (AS-OCT). For intraocular pressure (IOP), all the rabbits were examined with Tonopen tonometer before and after modeling, and IOP of the rabbits before modeling and from control group was used as the control. Five eyes of the rabbits from experimental group we rerespectively enucleated for subsequent examination at 1 week, 1 month, 2 and 4 months postoperatively. After eyeball enucleation, the rabbits were then euthanized using a lethal dose of pentobarbital (60 mg/kg iv).

### Histological analysis

The rabbits’ eyes were enucleated and immediately fixed in 4% formaldehyde. The corneoscleral junction tissue (including 1 mm of corneal tissue from limbus, iris, and 1 mm of the tissue back from limbus) was isolated and processed for paraffin embedding. 4μmsections were stained with H&E and observed under a microscope (model BH2RFL-T3, Olympus Corporation, Tokyo, Japan). The process of H&E staining was listed as follow.The slides were first incubated in a 60 °C oven for 30 min. Subsequently, they were dewaxed using xylene I for 15 min and xylene II for 15 min, hydrated with absolute ethanol for 10 min, 90% ethanol for 5 min, 85% ethanol for 5 min,80% ethanol for 5 min, 70% ethanol for 5 min, and then in two successive 1xPBS solutions. Following rehydration, slides were immersed in 10% hematoxylin for 2.5 min, differentiated with 1% hydrochloric acid and ethanol for 3 s, and stained with 0.5% eosin for 1 min.Then they were dehydrated in an alcohol gradient, permeabilized with xylene, and mounted. The slides obtained from the rabbit’s eyes of the control group were used as the control.

For immunofluorescence, primary antibodies were mouse anti-rabbit MMP2 antibody (1:100; abcam) and Mouse anti-rabbit AQP-1 antibody (1:100; Novas). Slices were incubated with the primary antibodies overnight at 4 °C. Secondary antibodies (1:100; obtained from Beijing Zhongshan Technologies) coupled to FITC or TRITC were then applied for detection.Subsequently, the slices were stained with DAPI (Solarbio life sciences, Beijing, China) to visualize the nuclei. Fluorescence was observed using a fluorescent microscope (model BH2RFL-T3, Olympus Corporation, Tokyo, Japan). Slices stained withphosphate buffer solution instead of primary antibody and those obtained from the eyes of the rabbits from control group were used as control. Other procedures were the same as the experimental group.

### Quantitative reverse-transcription polymerase chain reaction (qRT-PCR)

Two rabbits’ eyes were respectively enucleated on post-operative 1 week, 1 month, 2 and 4 months, and three eyes of three rabbits from the control group were used as control, Total RNA was extracted from the corneoscleral junction tissue in the angle-closure area with TRIzol reagent (Invitrogen Corporation, Carlsbad, CA, USA). The normal rabbits’ eyes were used as control group. Reverse transcription reactions were performed as follow: cDNA was synthesized with a First Strand cDNA SynthesisKit (Toyobo, Osaka, Japan) according to the manufacturer’s protocol. Then, qRT-PCR wasperformed in triplicate on a sequence detection system (ABI Prism 7000; Life Technologies/Applied Biosystems, Inc., Foster City, CA, USA). The mean CT values were calculated, and therelative expression values were calculated from the delta CT values using the formula: 2^-ΔΔCT^.The volume of RT-PCR was 20 μl, including 2 μl cDNA, 10 μl SYBR Green Real-time PCR Master Mix (Toyobo, Osaka, Japan), 1 μl each of specific forward and reverse primers, and 6 μlsterile water. Quantitative RT-PCRs were run in duplicate using a LightCycler (Applied Biosystems, Life Technology, USA) at 95 °C for 30 s, followed by 40 cycles of 95 °C for 5 s, 56–60 °C for10 seconds, and 72 °C for 60 s.The primers used were as follows: MMP2, forward: 5′-GAAGGTCAAGTGGTCCGTGT-3′, reverse: 5′-CCGTACTTGCCATCCTTCTC-3′); AQP1, forward5’- ACCACTGGATCTTCTGGGTG-3′; and reverse 5′- CATCTCCACCCTGGAG TTGA- 3′; Tissue inhibitors of metalloproteinase-2 (TIMP-2), forward: 5′- AAGCGGTCAGTGAGAAGGAAG -3′; and reverse 5′- GGGGCCGTGTAGATA AACTCTAT -3′. For thenormalization of the gene expression levels, the gene-to-GAPDH (housekeeping gene) was calculatedand compared to that of the normal rabbits.GAPDH forward:5’-GCGCCTGGTCAC CAGGGCTGCTT-3′; and reverse 5’-TGCCGAAGTGGTCGTGGATGACCT-3′. LightCycler software and Light Cycler Relative Quantification software were used to analyze the data.

### Enzyme-linked immunosorbent assay (ELISA)

Freshcorneoscleral junction tissue from the angle-closure area of the rabbits, about 30 g, was placed in ice-cooled EP tubes and homogenized in 500 μl phosphate buffered saline (PH = 7.4) containing 2% protease inhibitor (Roche, Indianapolis, IN) using hand held tissue grinding instrument. Three eyes of three rabbits from the control group were used as control. The content of MMP2 and TIMP2 were measured with a commercial rabbit MMP2 ELISA Kit (Multi Sciences LTD., Hangzhou, China) and a rabbit TIMP2 ELISA Kit (Cusabio, Wuhan, China) according to the protocol provided by the manufacturer. The optical density of each well was determined using a microplate reader (Bio-Rad 680, Hercules, USA) set to 450 nm. Wavelength correction was set to 570 nm. The protein concentration for each sample was calculated according to the standard curve.

### Statistical analysis

All statistical analyses were performed using SPSS 13.0. Data are presented as the mean ± standard deviation. One-way analysis of variance (ANOVA) was used for statistical analysis. Differences were considered statistically significant at *p* < 0.05.

## Results

### Animals and clinical presentation

**Fig. 2:**
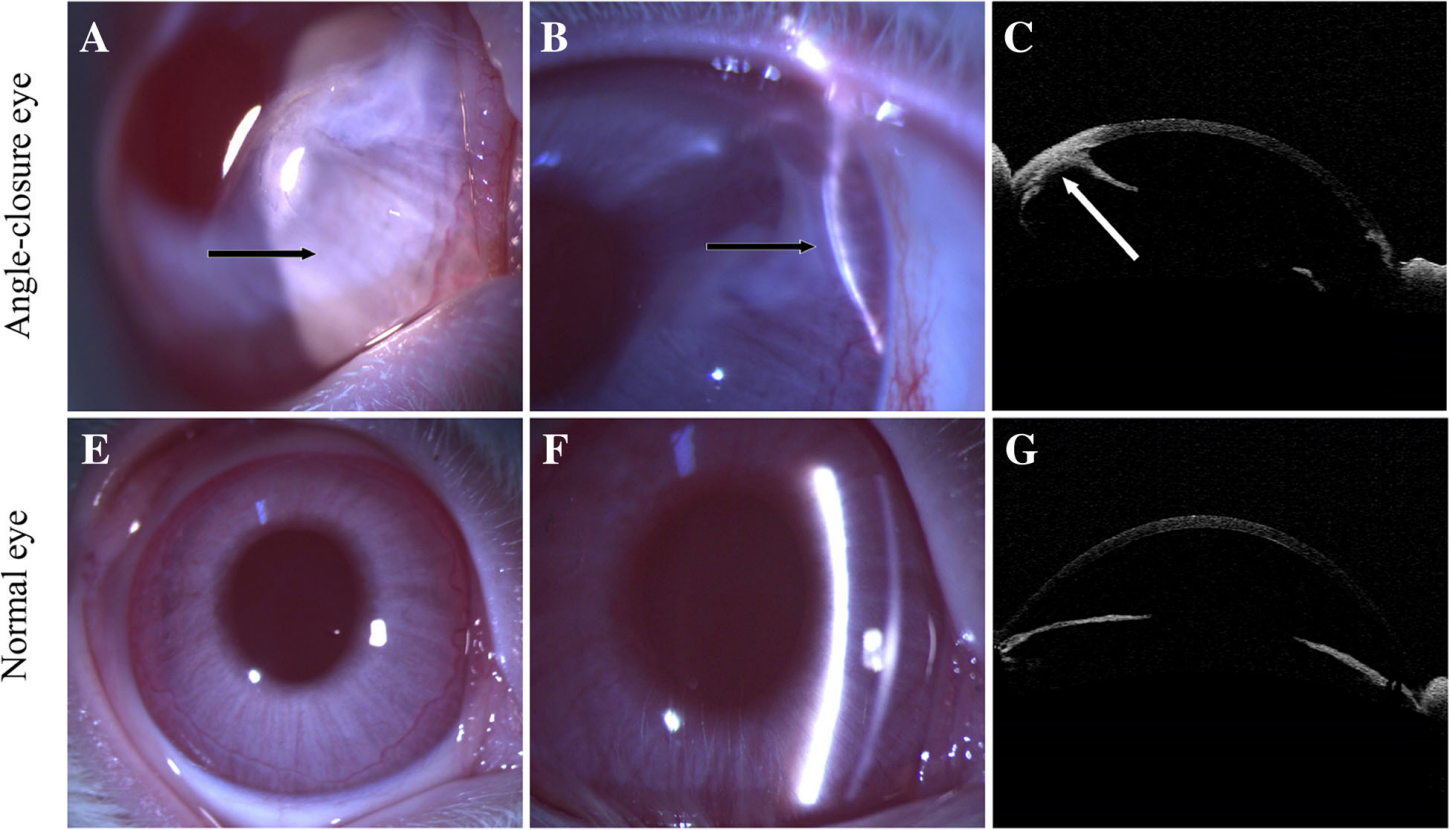
Clinical presentation of the rabbits. (**a-b**) Slit-lamp photography of the rabbit’s eye with angle-closure; (**c**) AS-OCT imagine of the rabbit’s eye with angle-closure; (**d**-**e**) Slit-lamp photography of the normal rabbit’s eye; (**f**) AS-OCT imagine of the normal rabbit’s eye.“→“showed the area of angle-closure

All animals survived without infections after establishment of angle-closure models. Angle-closure was observed in the eyes of twenty rabbits by slit-lamp examination (Fig. 2a and b), twelve rabbits undergoing one operation and eight rabbits subjected to twice. However, there still are five rabbits without angle-closure after repeated operation, and these rabbits were excluded in this study (data no shown). AS-OCT also verified the formation of angle-closure in the rabbits (Fig. 2c).

**Fig. 3:**
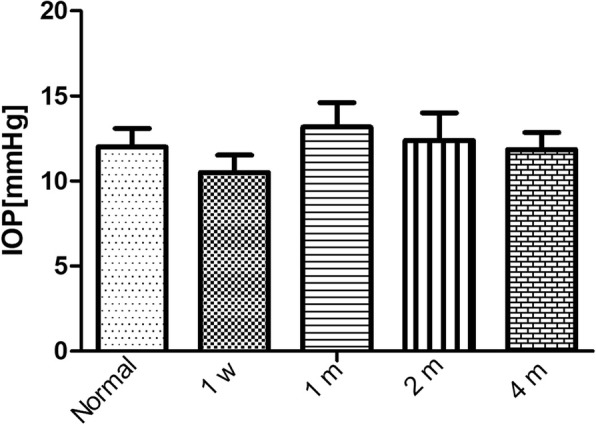
The intraocular pressure of the rabbits on pre-operation and 1 week, 1 month, 2 and 4 months after angle-closure. Data represent as mean ± SD of three rabbits with four repeated measurements

The IOP of all the rabbits distributed from 8.57 to 15.25 mmHg, and no significantly high IOP was found in the follow-up period. It also had no significant difference in IOP of the rabbits among pre-operation, post-operative 1 week, 1 month and 4 months (Fig. 3). The extent of angle-closure in all the rabbit models was very small, about 2 to 4 clock hours. The remaining normal chamber angle may function well to maintain normal IOP of the rabbits. We speculate that it may be the reason for rabbits with normal IOP postoperatively.

### Histological analyses

**Fig. 4:**
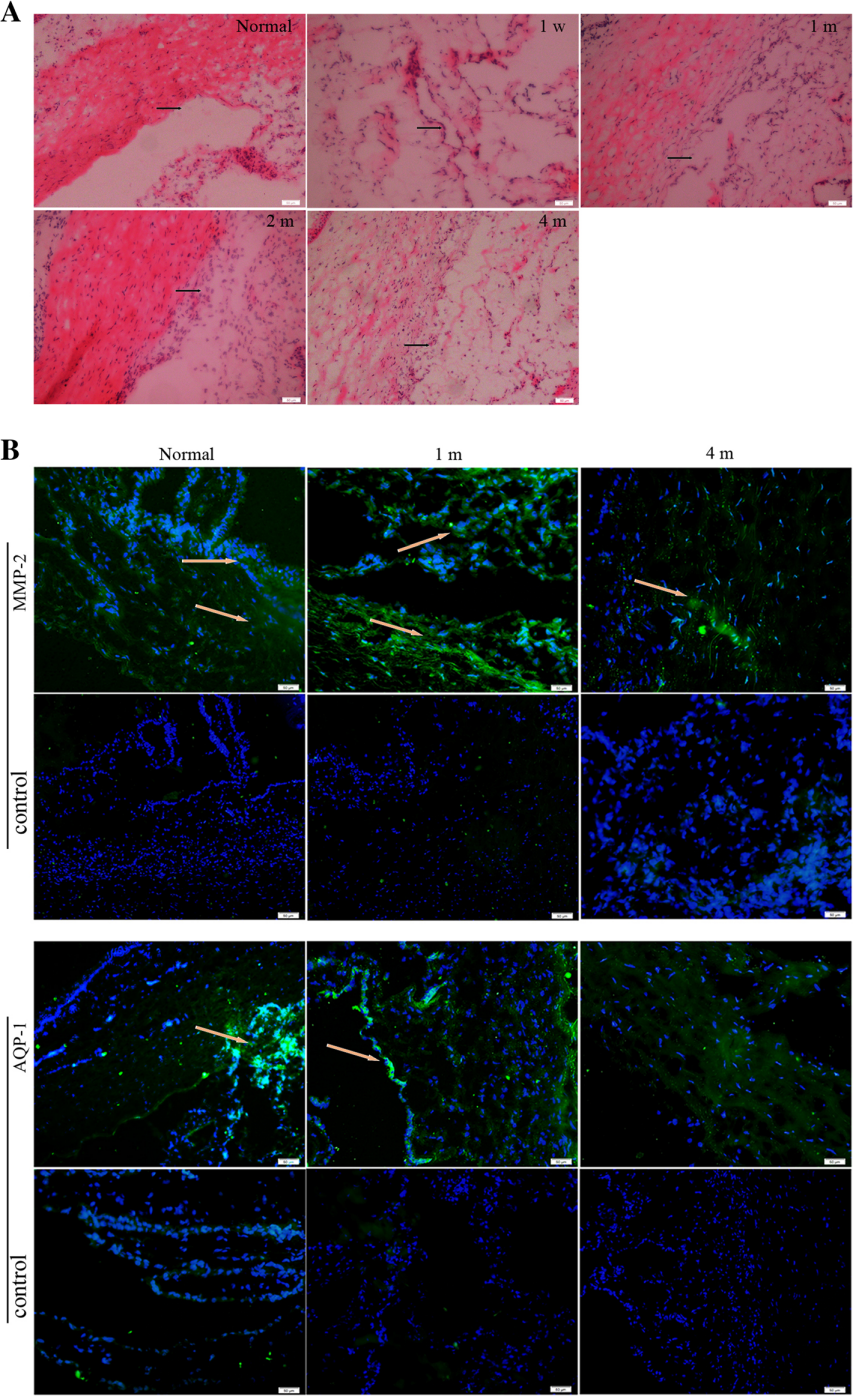
H&E stain and immunofluorescence images of the tissue of anterior chamber angle. (**a**) HE stain of the tissue of anterior chamber angleon pre-operation and 1 week, 1 month, 2 and 4 months after angle-closure.“→“showedthe anterior chamber angle of the rabbits’eyes; (**b**) immunofluorescence images of MMP-2 and AQP-1 in the tissue of anterior chamber angle on pre-operation and 1 week, 1 month, 2 and 4 months after angle-closure. Scale bar, 50 μm

H&E staining showed that the iris was attached to the inner surface of the corneoscleral junction, which also confirmed the angle-closure in the rabbit eyes (Fig. 4a). Immunohistochemical expression of AQP-1 and MMP2 was assessed in the corneoscleral junction of rabbit eyes. AQP-1 was mainly expressed in the cells located in Schlemm’s canal, the inner surface of TM, and the surface of the iris in both angle-closure group and control group. The staining was a little weaker in the angle-closure area compared to the control group, and the number of the AQP-1-positive cells began to decline on 1 month after angle-closure (Fig. 4b). MMP2 staining was diffuse in TM and iris. Immunofluorescence signal of MMP2 was stronger in the eyes with angle-closure than that in the normal rabbit’s eyes on postoperative 1 month, and then gradually weaken (Fig. 4b).

### qRT-PCR analysis

**Fig. 5:**
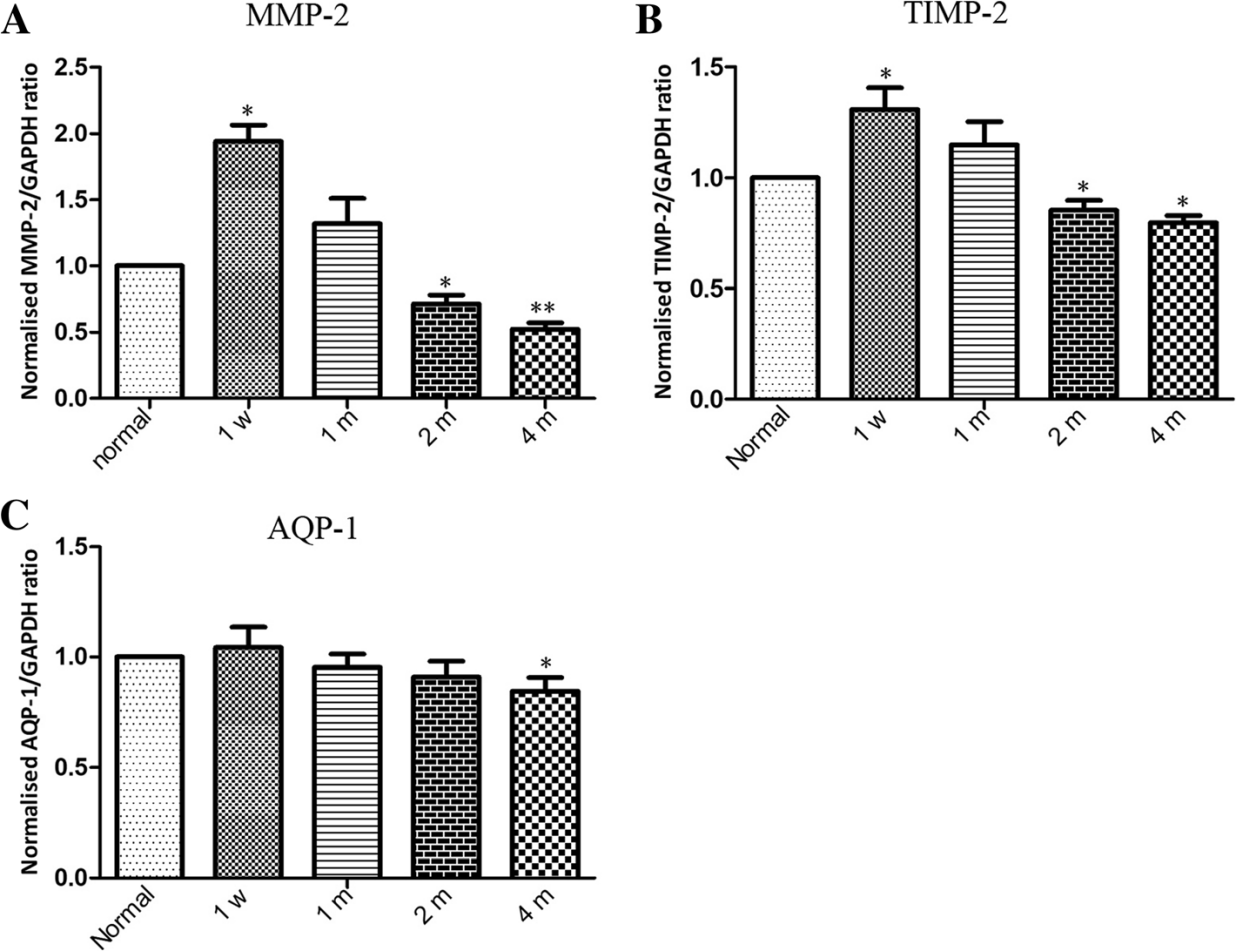
Time course of MMP-2, TIMP-2, and AQP-1 gene levels in the rabbit’s eyes after angle-closure (pre-operation and 1 week, 1 month, 2 and 4 months after angle-closure). Time course ofMMP-2 (**a**), TIMP-2 (**b**), and AQP-1 (**c**) gene.Data are representative of three replicatesof qRT-PCR and presented as the mean ± SD.One-way ANOVA was used for statistical analysis.*p < 0.05, **p < 0.01

qRT-PCR analysis was performed to detect the levels of MMP2 and AQP-1 after angle-closure. The concentration of RNA used in this study was 350-750 μg/ml. Compared with the control group, the expression of MMP2 was slightly upregulated on postoperative 1 week (averaged 1.94-fold) (*p* < 0.05), and then showed continuous downregulation in the follow-up period (Fig. 5a). There was no significant difference in the expression of AQP-1 between the angle-closure eyes and the controls within 2 months after angle-closure. On 4 months after angle-closure, AQP-1 levels slightly decline in the angle-closure eyes (*p* < 0.05) (Fig. 5c). TIMP-2 is the inhibitor of the MMP-2, and it also involved in the activation of MMP-2 [15]. Previous study showed that imbalance between MMPs and TIMPs may result in a change in ECM accumulation and cause increased aqueous humour outflow resistance [16]. Therefore, TIMP-2 gene levels were also detected in this study. We found that TIMP2, similar to MMP2, showed an increasing expression within postoperative 1 week, and slightly downregulated on 2 and 4 months after angle-closure (Fig. 5b).

### Expression of MMP2, TIMP2, and AQP-1 protein in corneoscleral junction

**Fig. 6:**
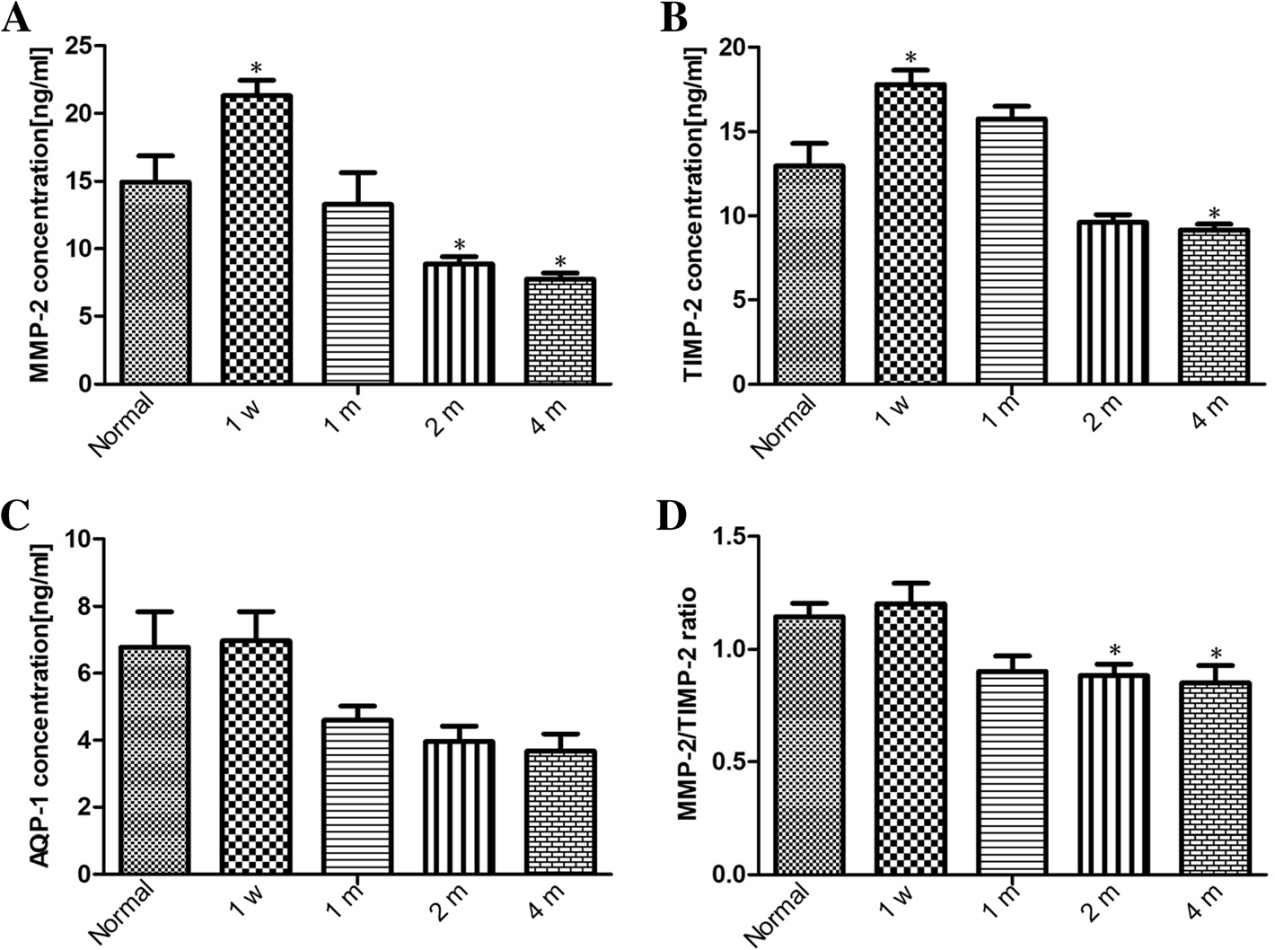
The changes of MMP-2, TIMP-2, and AQP-1 protein expression and MMP-2/TIMP-2 ratio in corneoscleral junction of the rabbit’s eyesbefore and after angle-closure.The changes of MMP-2 (**a**), TIMP-2 (**b**), and AQP-1 (**c**) protein expressionin corneoscleral junction of the rabbit’s eyesbefore and after angle-closure; The changes ofMMP-2/TIMP-2 ratio in corneoscleral junction of the rabbit’s eyes before and after angle-closure (**d**). Data are representative of three replicates of ELISA and presented as the mean ± SD. One-way ANOVA was used for statistical analysis.*p < 0.05

ELISA showed that compared to the control group, MMP-2 and TIMP-2 levels were significantly higher in the rabbit eye with angle-closure on postoperative 1 week (both *p* < 0.05), and slightly declined on 1 month, but the difference was not significant between the postoperative 1 month and control groups (both *p* > 0.05). Then, the expression of MMP-2 exhibited continuously decreased in the following 2 months. TIMP-2 levels were slightly declined on postoperative 2 months, but there was no significant difference compared to control group (p > 0.05). On postoperative 4 months, the expression of TIMP-2 was significantly lower than control group (*p* < 0.05)(Fig. 6a and b). Within 1 month after angle-closure, no significant change was found in the expression of AQP-1 between angle-closure and control eyes, and it slightly decreased on postoperative 2 and 4 months, but there was no significant difference compared to the control group (Fig. 6c).

Within 1 month after surgery, we did not find significant difference in the MMP-2/TIMP-2 ratio between the angle-closure and control eyes (p > 0.05). However, the ratio of MMP-2/TIMP-1 significantly declined on postoperative 2 and 4 months compared to the control group (*p* < 0.05) (Fig. 6d).

## Discussion

Permanent closure of the angle due to peripheral anterior synechiae (PAS) is the main cause of chronic angle-closure glaucoma and induces an increase in IOP [17]. Goniosynechialysis (GSL) can separate peripheral anterior synechia (PAS) from the angle, expose the functional TM, and therefore restoring its filtering function [18, 19]. TM physiology related study shows that this tissue has unique morphologic and functional properties involved in the regulation of aqueous humour outflow [20]. IOP-lowering effect of GSL was reduced in the eyes with long-standing PAS due to trabecular damage. Increased synthesis and deposition of ECM proteins in the TM is associated with TM dysfunction and IOP elevation in glaucoma [14]. MMPs have a vital role in ECM degradation and remodeling in the TM, which maintains the outflow pathway and IOP homeostasis [21]. AQPs present in cells of the TM and the endothelium of the Schlemm’s canal provide the channels for maintaining the folw of water under a gradient ofhydrostatic pressure in anterior chamber. AQP-1 is strongly expressed in endothelial cells of the TM and Schlemm canal [22]. Therefore, the MMP-2 and AQP-1 levels may reveal the change of the TM function after angle-closure.

MMP-2 belongs to the family of gelatinases because of their unique ability to degrade gelatin, which cannot degrade collagen I but can degrade fibronectin, laminins, and proteoglycans. These have a crucial role in ECM turnover and integrity in tissues and have been studied in glaucoma [21]. Previous study showed that PACG eyes also had higher levels of MMP-2 in aqueous humour compared to normal eyes [17]. In this study, we also found that MMP-2 level was significantly upregulated in the tissue of corneoscleral junction within 1 week after angle-closure. We speculate that this implies a more active ECM degradation and remodeling process in the area of angle-closure. AQP-1 had been reported to be expressed in the cells of the TM and Schlemm’s canal before [23]. Immunofluorescent staining showed that the number of the AQP-1- positive cells began to decline on 1 month after angle-closure. Meanwhile, the MMP-2 level downregulated in the area of angle-closure. We speculate that these mayimplytheexhaustion ofthe compensatory function of TM, and maybe TM damage occurredat thistime. However, theseproblems still need further investigation.

Regulation of aqueous humour outflow resistance is one of the key roles of TM, which is achieved by modification of ECM composition [24, 25]. TIMPs are potent non-selective inhibitors of active MMPs, thus it can inhibit the degradation of ECM in the TM and contribute to increased outflow resistance by reducing the activity of MMPs [24]. An imbalance between MMPs and TIMPs in aqueous humour samples has been suggested to be involved in the development of glaucoma [26]. In this study, the expression of TIMP-2 slightly increased on 1 week after angle-closure and downregulated in the following time. No significant difference was found in the MMP-2/TIMP-2 ratio between the angle-closure and control eyes within 1 month after angle-closure. These data suggest that in the early stage of angle-closure, ECM accumulation caused by elevated TIMPs levels may not function in the animal models that we established as in human primary angle-closure glaucoma. It was reported that TM cells are directly involved in the change of MMP and TIMP levels in the aqueous humour [24]. In our study, MMP-2/TIMP-2 ratio in the angle-closure eyes was significantly lower than that in controls on postoperative 2 and 4 months. We believe that it may be caused by the change of TM cells’ function after angle-closure. Furthermore, TIMP-2 is required for activation of MMP-2 by forming a ternary complex with pro-MMP-2 and MMP-14 [15, 27]. We speculate that the increased expression of TIMP-2 in the early stage of angle-closure may also take part in the activation of MMP-2 and maintain the function of TM and Schlemm’s canal. But the role of TIMP-2 in angle-closure still needs intensive study.

The method for the establishmentof angle-closure model in this study is simple and efficient, and angle-closure was well achieved in about 80% animals. However, the extent of angle-closure in all the rabbits was very small, about 2 to 4 clock hours, and the IOP of all animals was normal, which is different from the primary angle-closure glaucoma in human. Moreover, the angle-closure in this study was caused by ocular trauma. These may be a limitation of this study. For better illustration of these questions, we will investigate the difference between the angle-closure achieved in this study and primary angle-closure glaucoma in our future studies.

## Conclusions

In summary, our studies showed altered levels of MMPs, TIMPs, and AQP-1 in the angle-closure area, which could be involved in the damage of TM and Schlemm’s canal after angle-closure. Furthermore, we developed a feasible and effective method to establish rabbit angle-closure model, which may be suitable for the study on angle-closure.

## Abbreviations

AQP: Aquaporin
AS-OCT: Anterior segment optical coherent tomography
ECM: extracellular matrix
ELISA: Enzyme-linked immunosorbent assay
GSL: Goniosynechialysis
IOP: Intraocular pressure
MMP: Metalloproteinases
PAS: Peripheral anterior synechia
POAG: Primary open-angle glaucoma
qRT-PCR: Quantitative reverse-transcription polymerase chain reaction
TIMP: Tissue inhibitors of metalloproteinase
TM: Trabecular meshwork
